# Role of Pharmacogenetics in Adverse Drug Reactions: An Update towards Personalized Medicine

**DOI:** 10.3389/fphar.2021.651720

**Published:** 2021-04-30

**Authors:** Emanuele Micaglio, Emanuela T. Locati, Michelle M. Monasky, Federico Romani, Francesca Heilbron, Carlo Pappone

**Affiliations:** ^1^ Arrhythmology and Electrophysiology Department, IRCCS Policlinico San Donato, Milan, Italy; ^2^ Milano Bicocca University, Istituto Auxologico, Milan, Italy; ^3^ Vita-Salute San Raffaele University, (Vita-Salute University) for Federico Romani, Milan, Italy

**Keywords:** adverse drug reaction, long QT syndrome, brugada syndrome, seizure, diabetes, proarrhythmia, genetic test, personalized medicine

## Abstract

Adverse drug reactions (ADRs) are an important and frequent cause of morbidity and mortality. ADR can be related to a variety of drugs, including anticonvulsants, anaesthetics, antibiotics, antiretroviral, anticancer, and antiarrhythmics, and can involve every organ or apparatus. The causes of ADRs are still poorly understood due to their clinical heterogeneity and complexity. In this scenario, genetic predisposition toward ADRs is an emerging issue, not only in anticancer chemotherapy, but also in many other fields of medicine, including hemolytic anemia due to glucose-6-phosphate dehydrogenase (G6PD) deficiency, aplastic anemia, porphyria, malignant hyperthermia, epidermal tissue necrosis (Lyell’s Syndrome and Stevens-Johnson Syndrome), epilepsy, thyroid diseases, diabetes, Long QT and Brugada Syndromes. The role of genetic mutations in the ADRs pathogenesis has been shown either for dose-dependent or for dose-independent reactions. In this review, we present an update of the genetic background of ADRs, with phenotypic manifestations involving blood, muscles, heart, thyroid, liver, and skin disorders. This review aims to illustrate the growing usefulness of genetics both to prevent ADRs and to optimize the safe therapeutic use of many common drugs. In this prospective, ADRs could become an untoward “stress test,” leading to new diagnosis of genetic-determined diseases. Thus, the wider use of pharmacogenetic testing in the work-up of ADRs will lead to new clinical diagnosis of previously unsuspected diseases and to improved safety and efficacy of therapies. Improving the genotype-phenotype correlation through new lab techniques and implementation of artificial intelligence in the future may lead to personalized medicine, able to predict ADR and consequently to choose the appropriate compound and dosage for each patient.

## Introduction

Adverse drug reactions (ADRs) remain an important and frequent cause of worldwide morbidity and mortality (Bouvy et al., 2015). Despite the dramatic technical improvements in molecular diagnoses, the causes of ADRs are still poorly understood. ADRs can be due to a huge variety of drugs and can involve every organ or apparatus, and several ADRs have recently been associated with specific genetic mutations. In this scenario, genetic predisposition toward ADRs is an emerging issue, not only in anticancer chemotherapy, but also in many other fields of contemporary medicine, including glucose-6-phosphate dehydrogenase (G6PD) deficiency, malignant hyperthermia, epidermal tissue necrosis (Lyell’s Syndrome and Stevens-Johnson Syndrome), epilepsy, thyroid diseases, porphyria, aplastic anemia, Long QT Syndrome, and Brugada Syndrome (Swen et al., 2011; García-González et al., 2016; Weitzel et al., 2017). This awareness led to a well-differentiated subspecialty of clinical genetics called “pharmacogenetics,” defined as “the study of how variations in a few genes affect the response to medications” (Wilke et al., 2007; Daly 2017), aimed to prevent ADRs and to optimize therapies, considering the individual variations in either pharmacokinetics or pharmacodynamics. Both pharmacokinetics, which is the complex interplay of absorption, distribution, metabolism and excretion, and pharmacodynamics, which is the physiologic consequence of the interaction between drug metabolites and receptors, are genetically encoded (Martinez-Matilla et al., 2019). The family of genes called CYP encodes the vast majority of enzymes regulating both pharmacokinetics and pharmacodynamics (Dresser et al., 2000).

In our view, the main role of pharmacogenetics is to translate genetic information into everyday medical practice, trying to lower the impact of ADRs, both for patients and for the healthcare system. It is beyond the scope of this review to discuss the full array of pharmacogenetic variants, whereas its aim is to present an update on the genetic background of ADRs, and to provide some red flags useful in everyday clinical practice, showing how genetics can be helpful for the prevention and the treatment of patients experiencing ADRs. Wider use of genetic testing and implementation of artificial intelligence techniques in the future may favor personalized medicine (Leopold and joseph, 2018), able to predict ADR, and consequently to choose the appropriate and safer drug and dosage for each patient (Kalinin and Higgins, 2018; Schaik et al., 2020).

### Impact of Adverse Drug Reactions in Clinical Practice

In 2020, a systematic review of observational studies emphasized that, despite the negative clinical outcomes and high costs of ADRs, the lack of consistent definitions and good data collection methods limited the possibility to provide an accurate and comprehensive picture of the problem (Zhu et al., 2020). Nonetheless, according to this review, the cost per patient of preventable ADRs was measurable in thousands of dollars, associated with a considerably increased length of hospital stays (Sultana et al., 2013), with a cost estimate of over 30 billion dollars just in the United States (Formica et al., 2018).

However, the damage is not only economic, but consists of plenty of human lives lost. For instance, according to a meta-analysis published in 1998, more than 100,000 patients die yearly in the US because of ADRs (Lazarou et al., 1998). The most harmful ADRs involve the treatment of inflammatory, gastrointestinal, renal, and blood coagulation disorders. This is the reason why nonsteroidal anti-inflammatory drugs (NSAID), diuretics, anticoagulants, and antiplatelets have been recognized over time to be the major culprits, with prescribing errors being major contributors to the problem.

### Definitions of Adverse Drug Reactions

To understand how ADRs may affect the clinical practice, it is first necessary to define them. According to the World Health Organization (WHO) definition established in 1972, ADR can be defined as “a response to a drug which is noxious and unintended, and which occurs at doses normally used in man for the prophylaxis, diagnosis, or therapy of disease, or for the modifications of physiological function” (International Drug Monitoring: The Role of National Centres, 1972). According to the European Medicines Agency (EMA), “ADR is any untoward medical occurrence in a patient or clinical trial subject administered a medicinal product, and which does not necessarily have a causal relationship with this treatment” (Baldo, Francescon, and Fornasier 2018). However, in 2010 the European Union stated that the ADR definition should also encompass medication errors and off-label uses, and that there must be at least a reasonable possibility of causal relationship between drug and reaction (DIRECTIVE 2010/84/EU OF THE EUROPEAN PARLIAMENT AND OF THE COUNCIL of December 15, 2010). According to the Federal Drug Administration (FDA), ADR is “Any noxious, unintended and undesired effect of a drug, which occurs at doses used in humans for prophylaxis, diagnosis or therapy. This excludes therapeutic failures, intentional and accidental poisoning and drug abuse” (Wu et al., 2019). Besides, “adverse event means any untoward medical occurrence associated with the use of a drug in humans, whether or not considered drug related” (Sakaeda et al., 2013). Noteworthy, ADRs are caused by multiple mechanisms, and indeed we now distinguish two main types of adverse drug reactions (Edwards and Aronson 2000; Wilke et al., 2007) with possible implications for the therapeutic management:

• ***Dose-related reactions*** (***Type A reactions***) caused by an overdosing of the compound relative to individuals and resulting in either toxic effects or side effects. This type of ADR is predictable and related to the pharmacodynamics and pharmacokinetics of the drug, and it is usually associated with milder effects and thus with lower mortality. Both pharmacokinetic and pharmacodynamics properties are related to enzyme proteins, and thus may depend on genetics. Therefore, a recent field within genetics has been called “pharmacogenetics,” studying both variants and mutations related to drug metabolism, dealing with treatment and prevention of many ADRs. The most relevant clinical application of Pharmacogenetics is in anticancer chemotherapy (Lu et al., 2015), but this field is beyond the aims of this review.
• ***Non-dose related or idiosynchrasic reactions*** (***Type B reactions***), less common than dose-related reactions, generally considered unpredictable, although screening tests can indeed predict some of these reactions (Edwards and Aronson 2000). They can be caused by different mechanisms, often related to genetic conditions. Possible examples are malignant hyperthermia, porphyria, glucose 6 phosphate dehydrogenase deficiency, or drug-induced liver injury. The causes of non-dose related ADRs are not yet fully understood. ADRs can be caused by altered pharmacokinetics, due to altered absorption or metabolism, but also by altered pharmacodynamic properties, such as the alteration of the target receptor, or its pathway, or binding to unwanted receptors. Also, for non-dose related ADRs, both pharmacokinetic and pharmacodynamic properties are related to enzyme proteins, thus depending on genetics. However, the most likely mechanism for idiosyncratic reactions is an immunological cause (Uetrecht and Naisbitt 2013), for example related to mastocytes activity.

### Role of Genetic Codification in Adverse Drug Reactions

Several observations suggest that ADRs may run in families, although generally a Mendelian pattern of inheritance cannot be identified. Even in cases where genetic mutations are identified, these are only indicative of susceptibility to ADRs but cannot predict them with certainty (Wilke et al., 2007). Moreover, ADRs may happen in patients harbouring variants of uncertain significance (VUS), rather than pathogenic mutations or full syndromic clinical pictures (Pirmohamed, 2010). We provide here a synthetic glossary on the terms used in this review, and a more comprehensive compendium of pharmacogenetics nomenclature can be found in a recent article (Kalman et al., 2016). The nomenclature for genes in the text in provided in accordance with HUGO guidelines https://www.genenames.org/about/guidelines.

Genetic information is stored in complex molecules, such as genomic DNA, mitochondrial DNA (mtDNA), and various kinds of RNAs (Brenner, 2001). This information is organized in the genetic code, consisting of sixty-four nucleotides triplets, three stop signals, several regulatory sequences and regions interacting with both mtDNA and full RNA sets (Wang et al., 2009). All information stored in the genetic code specifies twenty canonical and two additional aminoacids (Ambrogelly et al., 2007), forming a huge variety of proteins. Proteins are the key-of-life, allowing countless activities in the cells, interacting with the environment to control and balance all aspects of cell life. Thus, the study of the genetic basis of ADR has to consider the complex interplay between the genetic information and environmental factors in a given disease condition (Afsar et al., 2019). Finally, it must be considered that the huge variability of genetic information is transmitted from one generation to the other, not always unaltered, inserting a further degree of variability, with possible great clinical relevance (Cismaru et al., 2020).

“Gene” is a DNA sequence occupying a precise position (called “locus”) in the genomic DNA itself. Every individual has two copies of non-sexual genetic information defined as “allele,” that can be considered alternative forms of a gene occurring at the same locus, each autosomic allele being inherited from either parent (García-González et al., 2016). The genetic sequence of every allele can be different from an individual to another. An allele is defined as “variant” when found with a frequency below 1% in the general population, excluding inbred coupling (Lo et al., 2003). In clinical settings, when a variant is associated to at least one specific pathologic phenotype, it is usually referred as a “mutation”. Instead, when a variant has a frequency above 1% in the general population, it is defined “Single Nucleotide Polymorphism (SNP)“ (Karki et al., 2015). SNPs are the most common contributors to variation in genetic code: copy number variants, insertions, deletions and duplications are the possible mechanisms. Not all SNPs have the same influence toward the risk of ADRs, however SNPs affecting regulatory RNAs have been recently described to have an important role in drug effects (Sadee et al., 2011). In pharmacogenetics, both relevant variants and SNPs are marked with the symbol “*” (star). A “haplotype“ is the order by which either single variants or SNPs are positioned across a gene (Sangkuhl et al., 2020). The relevance of haplotypes in pharmacogenetics is a direct consequence of the interactions between coding and non-coding genetic information.

Genes can encode one or more messenger RNA (mRNA), each mRNA allowing the synthesis of a protein or having a regulatory role (Portin and Wilkins 2017). The discovery of the huge amount of non-coding RNAs challenged the traditional definition of genes, demonstrating both the overlapping of many biological functions and the clinical relevance of genetic regulatory information (Gerstein et al., 2007). A recent review focused on the role of microRNAs (miRNAs), 18–22 nucleotide RNAs modulating the expression of multiple protein-encoding genes at the post-transcriptional level (Latini et al., 2019). MiRNAs modulate the pharmacological response, regulating numerous genes involved in the pharmacokinetics and pharmacodynamics of drugs, and differences in the levels of circulating miRNAs or genetic variants in the sequences of the miRNA genes can contribute to inter-individual variability in drug response, both in terms of toxicity and efficacy. For their stability in body fluids and the easy availability and accurate quantification, miRNAs could be ideal biomarkers of individual response to drugs (Latini et al., 2019).

### Role of Metabolism in Adverse Drug Reactions

Genetic mutations affecting both pharmacokinetics and pharmacodynamics can confer a predisposition toward ADR (Mehrotra et al., 2007). Probably, the prevalent mechanism affecting a drug behavior inside the body is related to the renal and hepatic metabolism (Doogue and Polasek 2011; Talal et al., 2017). If these two systems are impaired, the direct consequence will be a defective clearance of all compounds relying upon renal and hepatic metabolism. Besides, other disease conditions, including congestive heart failure, diabetes, peripheral vascular, pulmonary, rheumatologic, and malignant diseases are associated with increased ADR (Zhang et al., 2009).

Furthermore, other important modulators affecting the predisposition to ADRs are the immunologic factors. There are four main types of immunological reactions to drugs, namely the immediate hypersensitivity IgE-mediated, IgG-mediated, serum sickness, and delayed type cell-mediated (Dispenza, 2019). Also, immunological and genetic factors can interact in the pathogenesis of ADRs, through specific alleles in HLA-genes (Ferner and Aronson, 2019). Covering the role of modulators is beyond the scope of this review. Noteworthy, the modulating factors involved in drug metabolism may interplay not only with a disease specific mutation, but also with other environmental factors, such as age and pollutants.

### Environmental, Age and Gender Factors in Adverse Drug Reactions

The effect of both environmental and age and gender factors in drug metabolism is a well-described phenomenon. Several studies describe the relationship between age and drug metabolism (O’Mahony and Woodhouse, 1994; Benedetti et al., 2007). Pediatric age (Gregornik et al., 2021) and, even more so, geriatric age are at higher risk for ADRs (Seripa et al., 2015). Geriatric patients at higher risk for ADRs as they take several drugs, with possible mutual metabolic interferences. Moreover aging can lead to impaired biotransformation of several drugs, especially for decreased activity of many cytochrome enzymes (Wauthier et al., 2007).

The gender factor in ADRs is often underestimated, even if gender differences in drug metabolism may be a cofactor of ADRs. Indeed, men and women have a different chromosome, translating into diverse hormonal, metabolic, structural and immunological states. In general, women are at higher risk for ADRs, in particular when treated with thyroid hormones, TNF-alpha inhibitors and analeptics (Tran et al., 1998). Also, the risk of pro-arrhythmic effect of several cardiac and non-cardiac drugs is higher in women (Roden, 2016).

Besides age and gender, other environmental factors may affect ADRs, particularly cigarette smoking and pollutants, which can modify CYP1A1 and CYP1A2 expression (Wardlaw et al., 1998). This might alter the pharmacokinetics of many drugs, such as haloperidol, propranolol and flecainide. Viral infections can likewise change the behavior of a drug and induce an adverse drug effect, namely with ampicillin rash in infectious mononucleosis, Reye’s syndrome in patients with Influenza B and Varicella Zoster viruses and multiple drugs with HIV positive patients due to their altered metabolism (Levy, 1997).

### Adverse Drug Reactions in Different Disease Conditions

Genetics has been known to affect drug metabolism since the discovery of glucose-6-phosphate dehydrogenase (G6PD) deficiency. This condition is also known as “favism” because affected patients can experience hemolytic anemia after a meal with fava beans. Over time, researchers have found associations between hemolytic anemia and the consumption of other foods (like beans or peas), or the use of certain drugs (Beutler, 2008). Other classical conditions related to ADRs are malignant hyperthermia, Lyell syndrome, Stevens-Johnson Syndrome, porphyria, Fanconi Syndrome, Long QT Syndrome, and Brugada Syndrome (Swen et al., 2011; García-González et al., 2016; Weitzel et al., 2017). For most of them, a genetic background has been defined, especially following the technical improvements of genetic testing due to Next Generation Sequencing (NGS) (Hwang et al., 2017; Yohe and Thyagarajan, 2017). This new knowledge brought two important pieces of clinical information: different kinds of ADRs can share the same genetic background (Hwang et al., 2017), and even common genetic polymorphisms might play a relevant role in the aetiology of ADRs, especially in elderly patients (Just et al., 2017).

### Drug Induced Haemolytic and Aplastic Anaemias

The most relevant drug-induced anaemias are haemolytic, sideroblastic or aplastic anaemia. The most common syndrome causing drug-induced haemolytic anaemia is the glucose-6-phosphate dehydrogenase (G6PD) deficienc*y* (Pamba et al., 2012; Luzzatto et al., 2016; Belfield and Tichy, 2018).

### Glucose-6-Phosphate Dehydrogenase Deficiency

In humans, G6PD deficiency is the most common enzyme deficiency due to an X-linked mutation associated with reduced G6PD enzymatic activity (Beutler 2008; Belfield and Tichy, 2018). This enzyme is involved in the pentose phosphate pathway, through which NADPH is produced. NADPH is required to protect human cells from oxidative stress, and when its concentration decreases, many tissues suffer from its impaired function. This is more evident in tissues with frequent cell divisions, like red bone marrow, in which erythrocytes mature and become functional. G6PD deficiency affects almost half a billion people all over the world. Due to the position of G6PD in the X chromosome, males can display two possible genotypes: wild type (meaning “non carrier”) or affected carrier. In females, instead, there are three possible genotypes: homozygous (affected), hemizygous (either affected or non-affected) and wild type (unaffected). The enzymatic deficit can be diagnosed by the detection of enzymatic activity with different methods. Noteworthy, most individuals with G6PD deficiency of either sex do not show a specific clinical picture throughout their life. However, exposure to either drugs or infection is sufficient to experience oxidative stress, responsible for the NADPH depletion, eliciting an acute haemolytic crisis. In these situations, symptoms can include jaundice, fatigue, back or abdominal pain, and haemoglobinuria (Beutler, 2008). Thus, G6PD deficiency should be included in differential diagnosis in case of patients affected by acute haemolysis after exposure to known oxidative drugs. A list of medications that should be avoided in patients with G6PD deficiency is provided in Table 1 complete list is available at www.g6pd.org. It is also possible to perform a molecular analysis to detect known mutations of the *G6PD* gene. This gene is characterized to be highly polymorphic in the general population, lacking mutational hotspots and with common missense mutations, regardless of ethnicity (Luzzatto et al., 2016). The two more frequent mutations are V68M and N126D, accounting for about half of the cases (Pamba et al., 2012). Other mutations can be found with a particularly high frequency in specific ethnic groups, such as in the Arab world (Doss Priya et al., 2016). Since the discovery of G6PD deficiency, many other syndromes and genetic variants have been associated with adverse drug reactions by linkage analysis and genome sequencing. Besides the G6PD deficiency, which will be further detailed among haemolytic anaemias, there are some other well-known genetic syndromes which are correlated to adverse drug reactions, with the most known being malignant hyperthermia.

**TABLE 1:** Drugs to be Avoided in Patients with Glucose-6-Phosphate Dehydrogenase Deficiency.

•**General medications**
anacin
empirin
excedrin
pepto bismol
acetylsalicilates
aspirin
bufferin
ecotrin
•**Antimalarials**
chloroquine
mefloquine
pamaquine
primaquine
quinidine
quinine
•**Sulfonamides and sulfones**
dapsone
furosemide
sulfacetamide
sulfamethoxazole
sulfanilamide
sulfasalazine
sulfisoxazole
nitrofurans
nitrofurantoin
•**Quinolones and fluoroquinolones**
ciprofloxacin
levofloxacin
moxifloxacin
norfloxacin
•**Other medications**
acetylphenylhydrazine
beta-naphthol
chloramphenicol
dimercaprol
fava beans
glyburide
menthol
penicillamine
phenazopyridine
phenylhydrazine
probenecid
rasburicase
tolbutamide

### Aplastic Anaemia

An example of drug-induced aplastic anemia can be found in patients with Fanconi Anemia (Mehta and Tolar, 1993; Auerbach, 2009). This is a genetic syndrome, usually autosomal recessive for B*RCA2*, *BRIP1*, *FANCA*, *FANCC*, *FANCD2*, *FANCE*, *FANF*, *FANCG*, *FANC*I genes, while autosomal dominant for *RAD51* or even X-linked for *FANCB* genes (Mehta and Tolar, 1993). The Fanconi anemia pathway comprises 19 gene products (FANCA to FANCT) (Ceccaldi et al., 2016). In most patients, typical congenital physical features, like short stature and skin pigmentation, besides bone marrow failure, characterize Fanconi Syndrome. This condition is the most common cause of adult-onset aplastic anemia, typically preceded by chemotherapy or chemo radiation, frequent therapies in those patients, due to their tendency to develop solid malignancies. Noteworthy, even the gene carrier status for pathogenic mutations in some Fanconi anemia genes (*FANCD2*, *FANCA*, *FANCB*, *FANCC*) has been associated with higher risk of bone marrow failure under replicative cell stress conditions, such as chemotherapy (Durrani et al., 2019).

Germline inactivation of any one of the Fanconi anemia genes causes Fanconi anaemia, resulting in sensitivity to interstrand-crosslinks (ICLs). ICLs are DNA lesions that inhibit essential processes, such as replication and transcription, and they must be repaired or bypassed for the cell to survive (Ceccaldi et al., 2016). Thus, ICLs predisposes patients to bone marrow failure and development of cancer.

In summary, individuals developing aplastic anaemia following chemo or radiotherapy should be suspected for Fanconi Syndrome and investigated for a possible carrier status, and once identified should undergo a careful oncologic and hematologic work-up, and avoid the most damaging chemo and radiotherapy, to preserve their bone marrow as much as possible (Mehta and Tolar, 1993).

### Porphyrias

Haemoglobin and myoglobin proteins are essential for life, both including a non-protein structure called “heme group” (Phillips 2019). The complex metabolic process to produce heme groups is linked to a specific enzyme pathway inside liver cells (Szlendak et al., 2016). Specific genes encode for each enzyme involved in the heme group pathway: so far, eight enzymes have been identified (Puy et al., 2010). Thus, there are eight known porphyria phenotypes, each caused by a specific genetic mutation: thus, porphyria is not a single disease, but a group of genetically different disorders, sharing some clinical manifestations. These disorders may be due either to altered levels or to altered activity of the enzyme encoded by a certain gene. The involved genes are *PBGD*, *ALAS*, *ALAD, UROS*, *UROD*, *CPOX*, *PPOX*, and *FECH* (Yasuda et al., 2019). It is relevant that all porphyria genes map on autosomes, while the only gene mapping on the X chromosome is *ALAS*, associated with a rare form of drug-related sideroblastic anemia (Furuyama and Sassa, 2000). This association is so characteristic that the onset of a sideroblastic anemia after a drug treatment is the main suspicion element for the erythropoietic porphyria (Phillips, 2019).

When an enzyme responsible for the heme group’s synthesis is deficient or defective, its specific substrate and all the other precursors (normally modified by that enzyme) can accumulate in the bone marrow, liver, skin, or other tissues, causing toxic effects. These precursors accumulate in the patient’s blood and are eliminated in the urine, bile, or stools (Balwani et al., 2017). This explain why the clinical pictures of all forms of porphyria can be characterized by hematologic manifestations (anaemia), neurologic disorders (sleep disorders, muscle weakness, seizures, chronic back, limbs, and abdominal pain), gastrointestinal disorders (constipation, diarrhoea, vomiting), and skin involvement (skin and mucosal ulcerations). Those signs and symptoms can be triggered not only by fever, viral infections (like C hepatitis), but also by certain drugs, especially anaesthetics and anticonvulsants. Table 2 provides a list of drugs associated with porphyria.

**TABLE 2:** Drugs associated with porphyrias.

•**Anaesthetics**
articaine
bupivacaine
lidocaine
mepivacaine
prilocaine
ropivacaine
•**Anticonvulsants**
carbamazepine
phenytoine
phenobarbitone
primidone
ethosuximide
tiagabine
felbamate
valproate
oxcarbazine

The most common forms of porphyria are acute intermittent porphyria (AIP) and porphyria cutanea tarda (PCT). Both forms can be diagnosed incidentally, often after puberty, since hormones are known triggers of accumulation of heme-group precursors in target organs (Haberman et al., 1975).

The pharmacogenetic relevance of porphyria is high, since several different drugs can trigger the clinical manifestations. The two most common forms of porphyria, AIP and PCT, have an identified genetic background: about 25% of PCT and 100% of AIP are inherited as autosomal dominant conditions. Thus, it is sufficient to harbor one mutated copy in either the *PBGD* or *UROD* gene to manifest the disease. Altogether, all forms of porphyria afflict fewer than 200,000 people in the United States, while in Europe the most common porphyria (PCT) has a prevalence of 1: 10,000 individuals, the other forms being much less common. The treatment of porphyria is beyond the scope of this study and is illustrated in detail elsewhere (Dawe, 2017).

The mechanisms of adverse drug reactions associated with porphyria are complex, likely due to competition between drug metabolizing enzymes and to enzyme dysfunctions inside liver cells (Bickers, 1982). Some forms of porphyria, especially AIP, show a specific association with anaesthetics like Articaine, Bupivacaine, Lidocaine, Mepivacaine, Prilocaine, and Ropivacaine (Nagarajappa et al., 2019). Moreover, porphyria can be diagnosed following the onset of skin lesions, like in Stevens Johnson syndrome, often exacerbated by sunlight exposure (i.e., photosensitivity). This commonly happens in patients harbouring single copy mutations in the *PBGD* gene. In other situations, an ADR due to psychotropic drugs like Valproic Acid (Noval Menéndez et al., 2012) can be the first sign. Noteworthy, a high incidence of psychiatric symptoms has been described in patients subsequently receiving a clinical diagnosis of porphyria (Santos et al., 2017). In summary, different variants of porphyria are metabolic disorders genetically determined, still difficult to diagnose. Their presence must be suspected in case of unexplained chronic abdominal pain (Valle Feijóo et al., 2015) or of adverse drug reactions against either anaesthetics or mood-modulating drugs. The relevance of genetic testing is growing, but at present there are no specific guidelines, unless a pathogenic gene mutation has already been found (Whatley and Badminton, 2013). Owing to many diverse clinical pictures, it is not possible, according to current knowledge, to perform genetic testing in porphyria as a first line diagnostic procedure (Stölzel et al., 2019). The only case in which genetic testing is strongly recommended is when a pathogenic mutation has already been found in the family (Whatley and Badminton, 2013). The pre-symptomatic genetic testing in those cases can be helpful to minimize the risk of porphyria acute attacks, in some cases very harmful.

### Malignant Hyperthermia

Malignant hyperthermia can be considered a pharmacogenetic disorder, which is most known for its sudden adverse reactions to certain drugs. Malignant hyperthermia is basically a metabolic muscle disease caused by heterozygous mutations in three genes to the best of current knowledge (Miller et al., 2018). The metabolic defect genetically encoded in this condition, regardless of the mutated gene, provokes a very fast and uncontrolled calcium increase from skeletal muscle cells (Riazi et al., 2018). It is common that a patient does not know they are affected by malignant hyperthermia until the administration of the triggering drugs (Wappler, 2010). Table 3 provides a list of drugs associated with malignant hyperthermia. The most harmful drugs for a patient affected by malignant hyperthermia are either volatile anaesthetics or succinylcholine (Horstick et al., 2013). Most of the individuals known to be affected by this syndrome are carriers of single copy mutations in the *RYR1* gene, located on chromosome 19q13.1, although rare mutations have also been found in the genes *CACNA1S* and *STAC3* (Horstick et al., 2013; Riazi et al., 2018). The incidence of this syndrome ranges between 1:10,000 and 1:250, 000 and the individuals affected develop a life-threatening adverse drug reaction upon administration of volatile anaesthetics such as halothane, sevoflurane, desflurane, and isoflurane. The signs of malignant hyperthermia include hyperthermia, tachycardia, tachypnoea, increased carbon dioxide production and oxygen consumption, acidosis, hyperkalaemia, muscle rigidity, and rhabdomyolysis, all related to a hypermetabolic response (Rosenberg et al., 2015). This kind of response is the main reason for the increasing body temperature that characterizes the clinical picture of such patients. In these cases, it is sufficient to avoid administration of the above-mentioned drugs, opting instead for intravenous anaesthetics. However, if the adverse reaction is already ongoing, a specific treatment consists of intravenous administration of Dantrolene, together with interruption of the causing drug and supportive therapy (Schneiderbanger et al., 2014). The knowledge of gene mutation causing malignant hyperthermia is crucial to avoid a potentially fatal anesthesia complication. Indeed, for over 30 years the diagnosis of malignant hyperthermia relied on *in vitro* functional tests, with a low negative predictive value (Loke and MacLennan, 1998). In suspected malignant hyperthermia (MH), the functional studies can have inconclusive results. This is due to the high frequency of polymorphic variants in RYR1 gene, the most commonly mutated gene in MH patients. Vice versa, genetic testing can provide clinically useful genotype–phenotype correlations (Zhou et al., 2007). These can be used in case of inconclusive results of a functional test. Thus, the introduction of genetic testing raised the negative predictive value up to 95% (Girard et al., 2004). Therefore, recently malignant hyperthermia became a rare condition, especially for family members of a proband, in whom a pathogenic mutation has already been identified. Notwithstanding, functional tests are still considered the gold standard for a definite diagnosis of malignant hyperthermia, either when a variant of uncertain significance is found, or when genetic testing results are negative (Yang et al., 2019).

**TABLE 3:** Drugs associated with malignant hyperthermia.

•**Depolarizing muscle relaxants**
succinylcholine (suxamethonium)
•**Inhaled general anaesthetics**
chloroform (trichloromethane, methyl trichloride)
desflurane
enflurane
halothane
Isoflurane
methoxyflurane
sevoflurane
Trichloroethylene
Xenon

### Drug Induced Cutaneous Reactions

Human skin is very often affected by adverse drug reactions, with highly variable clinical pictures (Chung et al., 2016; Lerch et al., 2018). The observation of a genetic background in patients affected by cutaneous ADRs was somewhat an unexpected finding (Gerogianni et al., 2018). Infact, the two major phenotypes of cutaneous ADRs are the **Lyell Syndrome**, also known as toxic epidermal necrolysis (TEN), and the **Stevens-Johnson Syndrome (SJS)** (Lerch et al., 2018; Oakley and Krishnamurthy, 2020). Variable muco-cutaneous lesions, displaying epidermal detachment and painful ulcerations in mouth, eyes, and genitalia, characterize both conditions. Such reactions are thought to be caused by an immunological response (Miliszewski et al., 2016). Both conditions share an onset characterized by a flu-like syndrome with fatigue and fever, besides either a neutrophilia or eosinophilia (Chung et al., 2016; Miliszewski et al., 2016). Furthermore, during the clinical course of both syndromes, immune deficiency can be diagnosed, often being the cause of death due to septic shock (Lerch et al., 2018). Currently, Lyell and Stevens-Johnson syndromes are considered as different expressions of the same clinical entity, sharing a similar genetic background, but mainly distinct by the grade of epidermal involvement. Specifically, if the area of detached skin is less than 10%, it is defined as SJS, while if it is more than 30%, it is defined as TEN, and if between 10 and 30% it will be considered an overlap syndrome (Banik et al., 2020). Of note, the drug dosage does not correlate to the severity of skin lesions, nor to their extension.

Genetic studies in Asian populations showed a correlation between HLA-B*1,502 and the two phenotypes (Chung and Hung, 2010), both correlating with ADRs following administration of carbamazepine (Fan et al., 2017; Wang et al., 2017). However, this correlation was not found in Caucasian populations, probably due to the lower prevalence of HLA-B*1,502 in those regions: therefore, US FDA now recommends testing for HLA-B*1,502 in Asian individuals before carbamazepine administration. Other HLA variants were found to be related to SJS/TEN are HLA-B*5,801 in Han Chinese patients and HLA-B*5,701 and HLA-A*3,101 in patients of European descent (Fan et al., 2017). Also, non-HLA-related genes have been linked to SJS/TEN such as CYP2C9*3, *GSTM1* and *TRAF3IP2* (Lerch et al., 2018). From a clinical standpoint, many drugs can cause such life-threatening adverse drug reactions, however the most common belong to aromatic anticonvulsants, such as carbamazepine (Wang et al., 2017). Other potentially harmful drugs are sulfonamides, allopurinol, and acetaminophen (Miller et al., 2018; Miliszewski et al., 2016; Ban et al., 2016). Table 4 provides a list of drugs potentially associated with TEN. These drugs are thought to bind to cellular peptide and type 1 Major Histocompatibility Complex (MHC), to create an immunogenic compound, resulting in a strong immune response (Miliszewski et al., 2016), leading first to nonspecific signs such as fever, malaise, and upper respiratory tract symptoms. Over the next few days, the patient develops a blistering rash with erosions, more localized in SJS, and more generalized in TEN (Kohanim et al., 2016; Miliszewski et al., 2016). Unfortunately, there is no specific therapy for this type of reaction. The patient should undergo supportive therapy to reduce the immune response, and lesion cleaning by antiseptics and antibiotics to reduce the risk of infection (Miliszewski et al., 2016). The strong association between specific HLA proteins stimulated further research to identify other possible risk or protective HLA alleles (Fan et al., 2017; Wang et al., 2017). A recent article published by a Japanese group also described single nucleotide polymorphisms in the *IKZF1* gene as a possible predisposing factor toward ocular involvement in SJS patients (Kohanim et al., 2016; Ueta, 2020). Further studies are required to confirm these results.

**TABLE 4:** Drugs associated with epidermal necrolysis (Stevens–Johnson and Lyell syndromes)

•**Sulfamidics**
Cotrimoxazole
Sulfasalazine
•**Penicillins**
Amoxicillin
Ampicillin
•**Quinolones**
Ciprofloxacine
Norfloxacine
Levofloxacine
Moxifloxacine
•**Anticonvulsants**
Lamotrigine
Valproic acid
Phenobarbital
Phenytoine
•**Miscellaneous**
Allopurinol
Piroxicam
Abacavir

### Drug Induced Epilepsy

The diagnosis of epilepsy is complex due to the variety of possible phenotypes and causes, including possible adverse drug reactions, involving either Central and/or Peripheral Nervous system, together with other organs (Godhwani and Bahna 2016; Božina et al., 2019). Indeed, drug-induced seizures are a common and dangerous consequence of several drugs, with more than 9% of all cases of status epilepticus being caused by drug or poison administration, usually antidepressants, stimulants, and antihistamines (Chen et al., 2016; Fricke-Galindo et al., 2018). Possible genetic predispositions toward drug-induced seizures is scarcely known, but since 2019, some experimental studies have shed new light on this hot topic. First, Wong and co-workers (Wong et al., 2019) studied the benefits of Donepezil administration in a murine model of Dravet syndrome. This condition is a common form of childhood onset epilepsy, caused by heterozygous mutations in the *SCN1A* gene. Their group also studied the role of HuperzineA (Hup-A), an acetylcholinesterase inhibitor, in the prevention of drug-induced seizures in *SCN1A* mutant mice, demonstrating the role of both muscarinic and γ-Aminobutyric acid type A (GABAA) receptors in Hup A-mediated seizure protection (Wong et al., 2016). The translation of these studies to the human species might be useful to prevent the side effects of anticonvulsant drugs in Dravet syndrome patients.

### Drug Induced Thyroid Diseases

Many drugs can cause thyroid dysfunction, either through a misbalance of the TSH-T3-T4 axis, or through an autoimmune mechanism. A common example of the latter is the autoimmune thyroid disease consequent to interferon-alpha treatment in patients with chronic Hepatitis C Virus (HCV) infection, also called interferon-induced thyroiditis (Hasham et al., 2013). Supporting a probable genetic predisposition of this condition is the linkage between variants in the genes *SP100*, *SP110*, *SP140*, *HLA*, and *TAP1* (Hasham et al., 2013). Also, polymorphisms affecting both UGT1A9 (UGT1A9-rs3832043) and nuclear receptor PXR (NR1I2-rs3814055, NR1I2-rs2472677, and NR1I2-rs10934498), possibly resulting in downregulation of liver metabolizing enzymes of Sorafenib (i.e., CYP and UGT), were associated with both plasma overexposure and severe thyroid toxicities upon Sorafenib intake in papillary thyroid cancer (Ba et al., 2020).

As most thyroiditis, also ADR-induced thyroiditis may start with an increase in thyroid hormones, then followed by a depression, usually permanent. For this reason, this reaction must be identified and treated promptly, or the patient will have to receive replacement thyroid hormones for their lifetime.

One of the most frequent ADR-induced thyroiditis is due to Amiodarone, a common antiarrhythmic drug (Ahmed et al., 2011). In the case of Amiodarone-related thyroid disorders, the mechanisms underlying AMIO thyroid toxicity have been elusive, and no link has been found yet between genetic variants and its thyroid disorders, both hypothyroidism and thyrotoxicosis (Jabrocka-Hybel et al., 2015). Noteworthy, some patients developing thyroid-related amiodarone adverse reactions are already affected by diverse thyroid disorders. Then, it is possible that variants impairing iodine metabolism may also modify the effects of iodine in amiodarone on the thyroid. Recently, it was suggested that Amiodarone treatment could induce endoplasmic reticulum (ER) stress in human thyroid cells, with possible implications of this effect in Amiodarone-induced destructive thyroiditis (Lombardi et al., 2015).

### Drug Induced Liver Diseases

Drug induced liver injury (DILI) is the leading cause of acute liver failure in Western Countries. Many studies have investigated the possible genetic predisposition in these events, mainly as to HLA-related genes. DILI is well documented in patients assuming amoxicillin and clavulanic acid, and specifically it has been observed that patients with increased susceptibility to this reaction carry HLA-DRB1*15:01, HLA-DQB1*06:02, HLA-A*02:01, and HLA-B*18:01 variants. However also other HLA variants have been found to be correlated with DILI, caused by Flucloxacillin, Minocycline, Lumiracoxib, Lapatinib, Ximelagatran, and Ticlopidine (Clare et al., 2017). Noteworthy, a genetic predisposition for non-alcoholic fatty liver disease (NAFLD) and Non-Alcoholic Steatohepatitis (NASH) has been observed, with the main player of these conditions being the variant I148M in the *PNPLA3* gene. Moreover, variants in the *TM6SF2, MBOAT7*, and *GCKR* genes have been found to be correlated with a moderate risk of NAFLD (Eslam et al., 2018). Therefore, in affected patients, the administration of substantial doses of multiple drugs mainly metabolized by the liver should be avoided, since hepatotoxic drugs may accelerate the NAFLD process.

### Diabetes and Adverse Drug Reactions

Many patients diagnosed with either type 1 or 2 diabetes are in reality affected by maturity onset diabetes of the young, also known as MODY (Valkovicova et al., 2019). This is a condition caused by a genetic mutation in any of the genes *ABCC8*, *APPL1*, *BLK*, *CEL*, *GCK*, *HNF1A*, *HNF1B*, *HNF4A*, *INS*, *KCNJ11*, *KLF11*, *NEUROD1*, *PAX4* or *PDX1*. The MODY phenotypes can be due to a variety of different genetic mechanisms, partially still not well understood but with possible implications for the therapeutic management. Regardless of genetic aetiology, a diabetic patient harboring such mutations might experience treatment failures with a frequency much higher than diabetics patients without those mutations (Chakera et al., 2015). Some forms are manageable just with diet, while others respond well to insulin and others to oral antidiabetic drugs (e.g., sulfonylureas and GLP-1 antagonists). In general, it is clinically relevant to assess the genetic origin of diabetes, as an erroneous therapy could lead to treatment failures, such as hyperglycaemic hyperosmolar syndrome or, inversely, severe hypoglycaemia (Bain, 2009).

### Proarrhythmic Drug Effects

Many different cardiac and non-cardiac drugs (mainly antiarrhythmic drugs) and non-cardiac drugs (including antihistaminic, antipsychotic, anti-depressant, antibiotics, antimalarial, gastrointestinal, and anaesthetic agents) have been described to favour the onset of fatal arrhythmias, defined as pro-arrhythmic effect (Roden and Anderson, 2006). Pro-arrhythmias may be due either to the prolongation of ventricular repolarization, defined as acquired long QT syndrome (LQTS), or to the induction of a Brugada pattern, unmasking Brugada Syndrome (BrS) (Roden and Anderson, 2006; Locati et al., 2017; Locati et al., 2019).

#### Drug-Induced QT Prolongation and Proarrhythmias

Many different cardiac drugs (*mainly antiarrhythmic drugs*) and non-cardiac drugs (*including antihistaminic, antipsychotic, anti-depressant, antibiotics, antimalarial, gastrointestinal, and anesthetic agents*) have been implicated in the prolongation of the ventricular repolarization and pro-arrhythmias (Locati et al., 2019; Roden, 2016) (Table 5). Virtually all QT prolonging drugs act by blocking the potassium channels, mainly the rapid component of the delayed rectifier potassium channel (I_kr_) encoded by the human Ether-à-go-go-Related Gene (*hERG*) (Antzelevitch and Burashnikov, 2011; Roden, 2016; Goversen et al., 2019; Locati et al., 2019; Roden, 2016). The complete and updated list of specific drugs that prolong the QT interval is available at www.qtdrugs.org. Some of these drugs have either been restricted or withdrawn from the market due to the increased incidence of torsade-de pointes (TdP), a particular polymorphic ventricular tachycardia typically associated with congenital or drug-induced prolonged QT interval.

**TABLE 5:** Cardiac and non-cardiac drugs associated with QT interval prolongation^a^.

**Cardiac medications**
**•**antiarrhythmic drugs
Class ia (Quinidine, procainamide, Disopyramide)
Class III (dofetilide, ibutilide, sotalol, amiodarone)
**Non-cardiac medications**
•antihistamines (*Terfenadine* ^b^, *Astemizole* ^b^)
•neuroleptic (*Haloperidol*, *Droperidol*, *Thioridazine*, *Chlorpromazine*)
•atypical antipsychotics (*Sertindole* ^b^, *Ziprasidone*, *Risperidone*, *Zimeldine*, *Citalopram*)
•antidepressants (*Amitriptyline*, *Imipramine*, *Maprotiline*, *Doxepin*, *Fluoxetin*)
•opiate agonists (*Methadone*, *Levomethadyl*)
•anesthetic agents (*Sevoflurane*, *Isoflurane*)
•antibiotics
•quinolones (*Sparfloxacin* ^b^, *Levofloxacin*, *Moxifloxacin*, *Grepafloxacin* ^b^)
•macrolides (*Erythromycin*, *Clarithromycin*, *Azitromycine*)
•antimalarials (*Quinine*, *Halofantrine*)
•immunosuppressants (*Hydroxychloroquine*, *Methotrexate*)
•antiprotozoal (*Pentamidine*)
•antifungal (*Azole group*)
•anti-motility agents (*Cisapride* ^b^, *Domperidone*)
•other (*Arsenic trioxide*, *Bepridil*, *Probucol*)

^a^ Complete and updated list of drugs can be obtained fromwww.qtdrugs.org or https://crediblemeds.org/

^b^Withdrawn from market or discontinued.

#### Reduced Repolarization Reserve and Proarrhythmias

**FIGURE 1:**
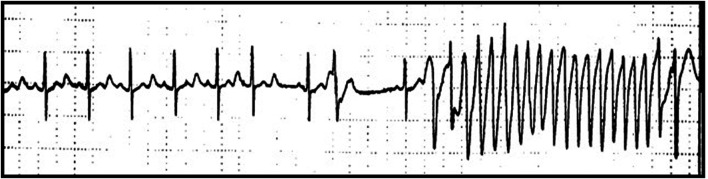
QT prolongation and Torsade-de-pointes (TdP) following Quinidine therapy. Marked QT prolongation (corrected QT, QTc 520 ms) and Torsade-de-pointes (TdP) following Quinidine therapy (dosage quinidine polygalacturonate 275 mg b. i.d, equivalent to quinidine sulfate 200 mg b. id.) recorded during Holter monitoring in a female patient (age 62 years) with history of paroxysmal atrial fibrillation.

Approximately 5–20% of patients with drug-induced TdP have mutations in genes causing LQTS. These patients have normal to borderline QTc interval at baseline but can develop QT prolongation and TdP, when exposed to I_kr_ blocking drugs (Roden, 2016). The risk of pro-arrhythmic effect is higher in women, in patients with structural heart disease, heart failure and electrolyte disturbance, due to use of diuretics or to renal or gastrointestinal diseases (Roden, 2016). The vulnerability to a pro-arrhythmic effect of a given drug is bound to the concept of “repolarization reserve” (Roden and Anderson, 2006). According to this theory, the loss of one component (such as rapidly delayed rectified potassium currents, I_Kr_) ordinarily will not lead to failure of repolarization (i.e., marked QT prolongation). However, individuals with subclinical lesions in other components of the system e.g., slowly delayed rectified potassium currents (I_Ks_) or calcium currents, may display little or no QT changes until I_Kr_ block is superimposed. Among antiarrhythmic drugs, ***Class IA agents*** (Quinidine, Procainamide and Disopyramide), blocking both Na^+^ and K^+^ channels (mainly I_kr_) can induce TdP either at therapeutic or subtherapeutic doses and can be precipitated by concomitant hypokalemia or hypomagnesaemia (Locati and Bagliani, 1999; Figure 1). On the other hand, ***Class III agents*** (Dofetilide, Ibutilide, Sotalol, Amiodarone), potent I_Kr_ blockers, prolong QT interval in a dose-dependent manner. Pro-arrhythmic effects may be favoured by concomitant factors, such as female sex, electrolyte imbalance, bradycardia, atrial fibrillation, or reduced left ventricular function, and the concomitant use of other QT prolonging drugs (Roden 2016; Locati and Bagliani, 1999; Frommeyer and Eckardt, 2016; Hreiche et al., 2008). In addition, several ***non-cardiac drugs*** (including antihistaminic, antipsychotic, anti-depressant, antibiotics, antimalarial, gastrointestinal, and anaesthetic agents) have been described to alter ventricular repolarization and favour arrhythmogenesis, by blocking the potassium channels, mainly the rapid component of the delayed rectifier potassium channel (I_kr_).

#### Sex Differences in Proarrhythmias

Women develop proarrhythmia with cardiac and non-cardiac dugs, and particularly with antiarrhythmic drugs, more often than men (Linde et al., 2018; Roden, 2016). Women have higher resting heart rates (HRs) and longer rate-corrected QT (QTc) intervals than men do, and sex differences in the electrical properties of cardiomyocytes and the myocardial conduction system have been extensively described (Curtis and Narasimha, 2012). Sex-based differences in clinical arrhythmias could be explained by changes in ion channel function, action potential (AP) morphology, autonomic tone, modulated by the effects of sex hormones and by genetics factors. Differences in the activity and density of ion channels, particularly K+ channels, in female cardiomyocytes may increase vulnerability to QT prolongation in women (Curtis and Narasimha, 2012). Several sex-based differences in function, structure, quantity, and currents of cardiomyocyte ion channels, including sodium (Na+), potassium (K+), and calcium (Ca2+) channels and their components, have been described. Sex hormones may affect Na + channel function and the transmural distribution of Na + channel current (INa), and particularly testosterone reduces the transmural dispersion in amplitude of Ina in males, while higher transmural dispersion of INa increases the risk of ventricular arrhythmias in females. Furthermore, a reduced expression of a variety of delayed rectifier K+ currents (IK) subunits was described in women compared with that in men (Curtis and Narasimha, 2012). Consequently, women have a higher risk than men for TdP in association with QT prolongation with class I and III antiarrhythmics (such as d,l-sotalol, amiodarone, dofetilide, and ibutilide), as well as with several non-cardiac drugs (see Table 5; Roden, 2016).

#### Factors Favouring Proarrhythmic Effects of Drugs

The pro-arrhythmic effect of a drug can be potentiated by the simultaneous use of multiple QT prolonging drugs, e.g., antibiotics, and antidepressants, or immunosuppressants (Roden, 2016). Drug-to-drug interactions have also been described, as several of these drugs are metabolized by the cytochrome P450 enzymes, CYP3A4 or CYP2D6 (Vos and Paulussen, 2004). Patients with liver dysfunction or co-administration of other drugs or food that inhibit the CYP3A4 or CYP2D6 can result in higher drug levels, and favor pro-arrhythmic effects (Roden, 2016; Zanger and Schwab, 2013; Tornio and Backman, 2018). Recently, during the COVID-19 pandemic, in order to provide an urgent remedy, multiple drugs by off-label use were administered to the patients, with a scarce knowledge of potential safety implications. Consequently, QT prolongation and cardiac arrhythmias have been described, particularly following the administration of hydroxychloroquine, an immunosuppressant derived from chloroquine, alone or in combination with antibiotics, such as azithromycin (Bernardini et al., 2020).

Also, impaired renal function may induce higher drugs and catabolite levels, favoring pro-arrhythmias (Reiffel and Appel, 2001). Furthermore, inherited salt-wasting renal disorders, such as Gitelman and Bartter syndromes, provoking hypokalaemia or hypomagnesemia, may favour pro-arrhythmias upon exposure to additional clinical stressors of repolarization, such as QT prolonging cardiac and non-cardiac drugs (Pachulski et al., 2005; Tsukakoshi et al., 2018).

**FIGURE 2:**
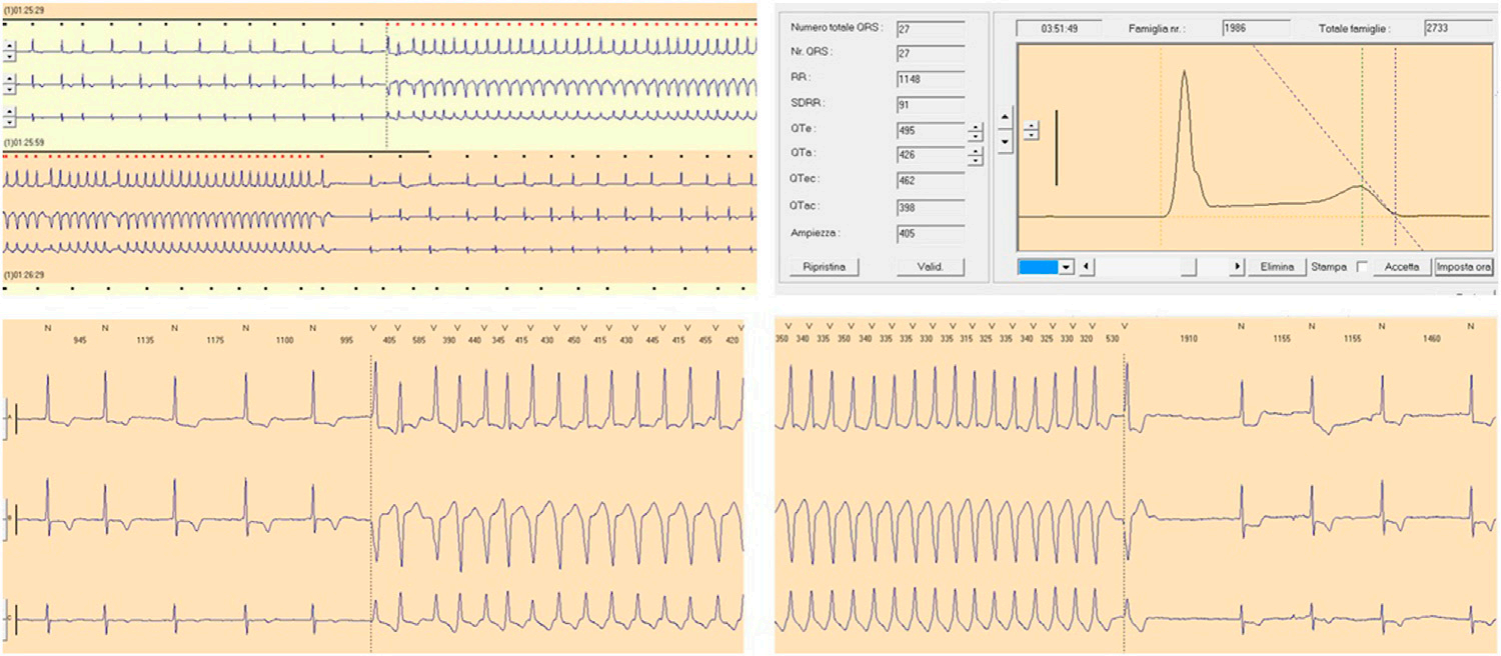
QT prolongation and ventricular tachycardia following Paroxetine therapy. Moderate QT prolongation (corrected QT, QTc 462 ms) and ventricular tachycardia following Paroxetine therapy (dosage 20 mg per day) recorded during Holter monitoring in a female patient (age 70 years) with a history of depression.

In summary, gene–drug interactions may also exist at different levels, favouring pro-arrhythmic effects of drugs (Figure 2):

• Rare ion-channel mutations that increase the risk of QT prolongation by drugs
• Common genetic variants that potentiate the QT prolonging effect of drugs
• Variation within drug metabolizing and transporting proteins that influence drug pharmacokinetics
• Simultaneous use of multiple QT prolonging drugs.

#### Congenital Long QT Syndrome and Proarrhythmic Drug Effects 

Among the cardiac syndromes known to be associated with adverse reactions to certain drugs, by far the most known is the **Long QT syndrome (LQTS)**. This syndrome is rather common, with an estimated prevalence from 1:2,000 to 1:20,000 (Schwartz et al., 1995; Schwartz et al., 2009). LQTS is characterized by a prolonged duration of ventricular repolarization, exposing affected individuals to a higher risk of fatal ventricular arrhythmias, such as torsade de pointes and ventricular fibrillation. Every patient diagnosed with this condition should receive the list of drugs further lengthening the QT interval, which must be avoided to prevent the occurrence of malignant arrhythmias (Fazio et al., 2013). A continuously updated list of QT prolonging drugs are available at the website https://crediblemeds.org/. Of the 17 genes reported as causative for LQTS, nine genes (*AKAP9*, *ANK2*, *CAV3*, *KCNE1*, *KCNE2*, *KCNJ2*, *KCNJ5*, *SCN4B*, *SNTA1*) were classified as having limited or disputed evidence as LQTS-causative genes. Only three genes (*KCNQ1*, *KCNH2*, *SCN5A*) were curated as definitive genes for typical LQTS. Another four genes (*CALM1*, *CALM2*, *CALM3*, *TRDN*) were found to have strong or definitive evidence for causality in LQTS with atypical features, including neonatal atrioventricular block, while the remaining gene (*CACNA1C*) had moderate-level evidence for causing LQTS (Adler et al., 2020; Zareba et al., 1998; Kutyifa et al., 2018). The three most common genotypes (LQT1, LQT2, and LQT3), accounting for most of LQTS cases, tend to have genotype-specific syncope triggers for cardiac events, characteristic T-wave morphologies, QT interval, and age and gender correlated features of high risk (Goldberg et al., 1991; Mathias et al., 2013; Giudicessi et al., 2018). Genotype specific age and gender differences have also been described (Zareba et al., 2003) Subjects with LQT1 and LQT2 tend to have several ‘‘warning” syncopal episodes before a sudden death, while in LQT3 the first presentation is commonly sudden death. Beta-blockers are the first-line therapy in LQT1 and LQT2 patients, while they have less efficacy in LQT3. Of note, in all genotypes, the strongest predictors for high risk are previous cardiac arrest or syncope and a QTc interval above 500 msec recorded at any time during follow-up (Locati, 2006).

• **LQT1** accounts for 40–55% of cases of the LQTS and is caused by mutations in the *KVLQT1* (also called *KCNQ1*) gene, encoding Iks. LQT1 patients typically show a prolonged QT interval and a broad-based T-wave on the resting ECG, and children and adolescents, predominantly male, are at highest risk, especially during exercise and particularly swimming.
• **LQT2** accounts for 35–45% of cases of congenital LQTS and is caused by a variety of mutations in the *hERG* (also known as *KCNH2*) potassium channel gene, encoding Ikr. The mutations may involve either the pore or the non-pore region of the hERG channel. Pore mutations carry high-risk of cardiac events and may affect young patients, whereas non-pore mutations often lead to TdP only in the presence of hypokalemia. In adult women with LQT2, particularly the post-partum period is potentially at highest risk for events. In LQT2, ventricular arrhythmias are typically observed during sleep, or during emotional or auditory stimulations. In addition to causing LQT2, the unique structural features of the tetrameric hERG/Kv11.1 channel make it particularly susceptible to blockade by an array of pharmacologic agents resulting in acquired or drug-induced LQTS (Roden, 2016; Frommeyer and Eckardt, 2016; Giudicessi et al., 2018).
• **LQT3** is caused by mutations in the sodium channel gene (*SCN5A*), and it is characterized by events occurring at rest or during sleep. LQT3 is due to perturbation of Na^+^-channel inactivation that can prolong the cellular action potential and alter cellular excitability, and it has the higher frequency of fatal cardiac events. In LQT3 patients, gene carriers are often bradycardic with very prolonged QT interval and late onset T waves on resting ECG, and typical T wave alternance can be observed before the occurrence of TdP, and it appears to be less responsive to beta-blockers.

#### Drug-Induced Brugada Pattern and Brugada Syndrome

**FIGURE 3:**
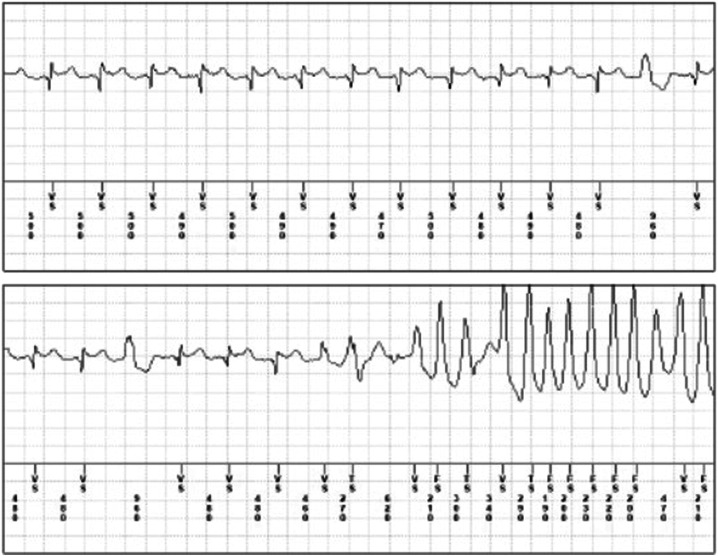
Brugada Pattern and ventricular tachycardia following flecainide therapy. Brugada Pattern and ventricular tachycardia following flecainide therapy (dosage 100 mg b. i.d) recorder by implantable loop recorder in a male patient (age 48 years) with a history of paroxysmal atrial fibrillation.

Another syndrome strictly correlated to drug-induced arrhythmias is **Brugada Syndrome (BrS).** BrS is characterized by a prolongation of ventricular depolarization mainly localized on the epicardial right ventricle outflow tract, typically manifested on the surface ECG by the elevation of the J point with a coved morphology defined as the Brugada pattern (Antzelevitch and Patocskai, 2016; Pappone and Santinelli, 2019; Priori et al., 2015). BrS is associated with a high risk of cardiac arrest and sudden death due to ventricular tachycardia and ventricular fibrillation. Typically, the Brugada pattern can be spontaneous or induced by sodium blocking drugs, like Ajmaline or Flecainide (Pappone and Santinelli, 2019; Turker et al., 2017; Figure 3). Thus, every patient diagnosed with BrS should avoid the use of sodium-blocker antiarrhythmics, and of several other drugs, including psychotropics, anaesthetics, and recreational drugs, such as alcohol and cocaine (Pappone and Santinelli, 2019; Table 6). The updated list of drugs associated with BrS is provided at http://www.brugadadrugs.org/. BrS is commonly considered a channelopathy, with an autosomal-dominant pattern of inheritance. The first gene variation associated with BrS was in the *SCN5A* gene, encoding the alpha-subunit of the cardiac sodium channel, Nav1.5, leading to a reduction of sodium currents (Ina) (Antzelevitch and Patocskai, 2016). Since then, more than 450 pathogenic variants have been identified in 24 genes encoding sodium, potassium, and calcium channels or associated proteins (*ABCC9, CACNA1C, CACNA2D1, CACNB2, FGF12, GPD1L, HCN4, HEY2, KCND2, KCND3, KCNE3, KCNE5, KCNH2, KCNJ8, PKP2, RANGRF, SCN10A, SCN1B, SCN2B, SCN3B, SCN5A, SEMA3A, SLMAP, and TRPM4*). Approximately 20–25% of BrS patients are genetically diagnosed with pathogenic variations in *SCN5A*, also accounting for the most severe clinical forms (Ciconte et al., 2020). However, known BrS susceptibility genes can only explain 30–35% of the clinically diagnosed cases, indicating that 65–70% of BrS patients remain genetically unsolved (Priori et al., 2015). Similarly as in drug-induced LQTS, latent forms of BrS can be induced or unmasked by a wide variety of drugs and pathological conditions. Of note, BrS is frequently associated with atrial fibrillation, whose therapeutic agents such class IC drugs (Flecainide and Propafenone) can unmask cases of latent or undiagnosed BrS (Pappone et al., 2009; Turker et al., 2017). Regarding the possible risk associated with anaesthetics, an important recent study conducted on 36 patients showed the safety of Propofol among high-risk patients clinically affected by BrS (Ciconte et al., 2018).

**TABLE 6:** Cardiac and non-cardiac drugs associated with Brugada Syndrome.

**Drugs able to unmask Brugada type 1 pattern, and to be avoided in patients with diagnosis of Brugada Syndrome.**
**•**Antiarrhythmic drugs*: Ajmaline, allapinin, ethacizine, flecainide, pilsicainide, procainamide, propafenone
**•**Psychotropic drugs: Amitriptyline, clomipramine, desipramine, lithium, loxapine, nortriptyline, oxcarbazepine, trifluoperazine anesthetics/analgesics*: bupivacaine, procaine, propofol
**•**Other substances: Acetylcholine, alcohol (toxicity), cannabis, cocaine, ergonovine
*For furthers advices please visit www.brugadadrugs.org/emergencies
**Drugs preferably avoided in patients with diagnosis of Brugada Syndrome.**
**•**Antiarrhythmic drugs: Amiodarone, cibenzoline, disopyramide, lidocaine**, propranolol, verapamil, vernakalant
**•**Psychotropic drugs: Bupropion, carbamazepine, clothiapine, cyamemazine, dosulepine, doxepin, fluoxetine, fluvoxamine, imipramine, lamotrigine, maprotiline, paroxetine, perphenazine, phenytoin, thioridazine
**•**Anesthetics/analgesics: Ketamine, tramadol other substances: Dimenhydrinate, diphenhydramine, edrophonium, indapamide, metoclopramide, terfenadine/Fexofenadine
**lidocaine use for local anesthesia (e.g., by dentists) does seem to be safe if the amount administered is low and if it is combined with adrenaline (epinephrine) which results in a local effect only
**Further recommendations**
**•**Recreational drugs (*alcohol, cocaine*) are also potentially dangerous in susceptible patients
**•**In case of fever, electrocardiographic monitoring is appropriate in combination with lowering of body temperature (e.g., by paracetamol/acetaminophen)
**•**Possible active drugs may be present in medications containing a combination of drugs
**•**The presence or absence of a specific drug on this list do not preclude a certain harmful or safe use of that specific drug in this patient respectively
**•**For most recent recommendations (and disclaimer) on drugs to be avoided by brugada syndrome patients, please visit http://www.brugadadrugs.org

## Discussion and Conclusions

Adverse drug reactions (ADRs) are often under evaluated and misdiagnosed, despite being an important cause of both morbidity and mortality all over the world. The technical improvement of genetic testing in the last years enabled the identification of multiple genes involved in ADRs. Thus, identifying the genetic risk factors particularly for idiosynchrasic ADRs could significantly decrease the morbidity and the mortality, and reduce healthcare costs (Wilke et al., 2007). Unfortunately, today the number of drugs for which it is possible to perform pharmacogenetic testing is still limited. The main clinical fields in which pharmacogenetic testing is already available are hemolitc anaemias, malignant hyperthermia, porphyrias, severe skin disorders, Brugada and long QT syndromes. Given the prominent role of genetics in pharmacokinetics and pharmacodynamics, an increasing use of pharmacogentic testing is likely to occur in the near future. Of note, today in case ADRs, the diagnostic work-up tends to consider environmental and genetic factors as mutually excluding, rather than interplaying. However, in reality ADRs could be viewed as a sign, a sort of “red flag,” of a possible genetic disorder. Those signs could be nonspecific (as Epidermal Necrolysis), or more related to specific genetic background (as QT interval prolongation and/or Brugada pattern). The clinical usefulness of “red flags” is double: first, raising the suspicion of an ADR, and second prompting a genetic counseling, particularly in case of familial recurrence. Thus, ADRs should become a new untoward “stress test,” which should raise the suspicion of a patient being carrier of a specific genetic background. In this context, the demonstration of a clinically relevant genetic background can optimize the safety of many common drugs, including anticonvulsants, anaesthetics, antibiotics, antiretroviral, and antiarrhythmics. The wider use of pharmacogenetic testing in the work-up of ADRs will have at least four consequences: 1) New clinical diagnosis of previously unsuspected diseases; 2) Improvement in the safety and efficacy of the therapeutic management, particularly in paediatric and elderly patients; 3) Repositioning of old drugs for new clinical indications, particularly in rare diseases; 4) Development of new drugs designed for certain specific genetic backgrounds. In this area, improving the genotype-phenotype correlation through new lab techniques and implementation of artificial intelligence in the future may lead to personalized medicine, able to predict ADR and consequently to choose the appropriate compound and dosage for each patient (Kalinin and Haggins, 2018) and the repositioning of old drugs for rare diseases (Scherman and Fetro, 2020).

## Abbreviations

AIP, acute intermittent porphyria; ADR, adverse drug reaction; BrS, Brugada syndrome; CYP, Cytochrome P450; DILI, drug induced liver injury; DNA, Deoxyribonucleic acid; GABAA, γ-aminobutyric acid type A receptors; G6PD, glucose-6-phosphate dehydrogenase deficiency; HLA, human leukocyte antigen; HCV, hepatitis C virus; LQTS, long QT syndrome; MH, malignant hypertermia; MHC, major histocompatibility complex; MODY, maturity onset diabetes of the young; mtDNA, mytocondrial deoxyribonucleic acid; mRNA, messenger ribonucleic acid; miRNA, micro ribonucleic acid; NAD, nicotinamide adenine dinucleotide; NADH, nicotinamide adenine dinucleotide hydrogenated; NAFLD, non-alcoholic fatty liver disease; NASH, non-alcoholic steatohepatitis; NGS, next generation sequencing; NSAID, nonsteroidal anti-inflammatory drugs; PCT, porphyria cutanea tarda; RNA, ribonucleic Acid; SJS, Stevens-Johnson syndrome; TdP, torsade-de pointes; TEN, toxic epidermal necrolysis.
